# White matter hyperintensity patterns: associations with comorbidities, amyloid, and cognition

**DOI:** 10.1186/s13195-024-01435-6

**Published:** 2024-04-01

**Authors:** Dario Bachmann, Bettina von Rickenbach, Andreas Buchmann, Martin Hüllner, Isabelle Zuber, Sandro Studer, Antje Saake, Katrin Rauen, Esmeralda Gruber, Roger M. Nitsch, Christoph Hock, Valerie Treyer, Anton Gietl

**Affiliations:** 1 Institute for Regenerative Medicine, University of Zurich, Campus Schlieren, Wagistrasse 12, 8952 Zurich, Schlieren, Switzerland; 2 Department of Health Sciences and Technology, ETH Zürich, 8093 Zurich, Switzerland; 3 Clinic for Aging Medicine, Hospital Affoltern, 8910 Affoltern, Switzerland; 4 Department of Nuclear Medicine, University Hospital of Zurich, University of Zurich, 8091 Zurich, Switzerland; 5 Department of Geriatric Psychiatry, Psychiatric Hospital Zurich, 8032 Zurich, Switzerland; 6 Neuroscience Center Zurich, University of Zurich, 8057 Zurich, Switzerland; 7 Neurimmune AG, 8952 Zurich, Schlieren, Switzerland

**Keywords:** Alzheimer’s disease, Small vessel disease, Amyloid-beta, Cognitive performance, Risk factors, Healthy aging, Clusters, Copathology

## Abstract

**Background:**

White matter hyperintensities (WMHs) are often measured globally, but spatial patterns of WMHs could underlie different risk factors and neuropathological and clinical correlates. We investigated the spatial heterogeneity of WMHs and their association with comorbidities, Alzheimer’s disease (AD) risk factors, and cognition.

**Methods:**

In this cross-sectional study, we studied 171 cognitively unimpaired (CU; median age: 65 years, range: 50 to 89) and 51 mildly cognitively impaired (MCI; median age: 72, range: 53 to 89) individuals with available amyloid (18F-flutementamol) PET and FLAIR-weighted images. Comorbidities were assessed using the Cumulative Illness Rating Scale (CIRS). Each participant’s white matter was segmented into 38 parcels, and WMH volume was calculated in each parcel. Correlated principal component analysis was applied to the parceled WMH data to determine patterns of WMH covariation. Adjusted and unadjusted linear regression models were used to investigate associations of component scores with comorbidities and AD-related factors. Using multiple linear regression, we tested whether WMH component scores predicted cognitive performance.

**Results:**

Principal component analysis identified four WMH components that broadly describe FLAIR signal hyperintensities in posterior, periventricular, and deep white matter regions, as well as basal ganglia and thalamic structures. In CU individuals, hypertension was associated with all patterns except the periventricular component. MCI individuals showed more diverse associations. The posterior and deep components were associated with renal disorders, the periventricular component was associated with increased amyloid, and the subcortical gray matter structures was associated with sleep disorders, endocrine/metabolic disorders, and increased amyloid. In the combined sample (CU + MCI), the main effects of WMH components were not associated with cognition but predicted poorer episodic memory performance in the presence of increased amyloid. No interaction between hypertension and the number of comorbidities on component scores was observed.

**Conclusion:**

Our study underscores the significance of understanding the regional distribution patterns of WMHs and the valuable insights that risk factors can offer regarding their underlying causes. Moreover, patterns of hyperintensities in periventricular regions and deep gray matter structures may have more pronounced cognitive implications, especially when amyloid pathology is also present.

**Supplementary Information:**

The online version contains supplementary material available at 10.1186/s13195-024-01435-6.

## Background

White matter hyperintensities (WMHs) are a very common finding in older adults and are associated with a greater risk of cognitive impairment [1, 2]. WMHs are typically interpreted as surrogates for cerebral small vessel disease, but the underlying pathophysiology is diverse and may also be related to non-vascular processes [3, 4]. Neuropathological, neuroimaging, and genome-wide association studies emphasize the multifactorial etiology of WMHs [4–7] and further suggest that the origin of WMHs varies depending on the brain region studied [4, 5]. Moreover, recent literature indicates that WMHs follow regional distribution patterns that are to some extent specific to disease and risk factors and present different neuropathologic associations as well as clinical consequences [2, 5, 7–9]. Thus, the identification of spatial patterns of WMHs and their corresponding phenotypical associations offer insights into regional risk and protective factors and may reveal patterns of increased clinical importance.

Studies exploring regional WMHs revealed that lesions exhibit regional associations with risk factors, sex, medical conditions, and cognitive symptoms [4, 7]. For instance, several studies found WMH patterns that are differentially associated with vascular risk factors and amyloid-β (Aβ) pathology. WMHs in posterior brain regions may be secondary to Alzheimer’s disease (AD) pathology, whereas WMHs in the deep white matter, particularly in the frontal regions, are more strongly associated with vascular risk factors such as hypertension [5, 10–12]. Although men typically exhibit a less favorable vascular risk factor profile, recent evidence indicates that in older individuals, women tend to have a greater total WMH volume [13], particularly after menopause [14], which may be attributed to higher WMH volume in deep brain regions [10, 15]. In terms of cognition, periventricular WMH burden generally exhibits a slightly stronger correlation with cognitive function than deep WMHs [16, 17], and increased posterior periventricular WMH volume has been observed in individuals with AD compared to those without cognitive impairment [18]. Additionally, evidence suggests that the co-occurrence of WMH and Aβ pathology increases the rate of cognitive decline in healthy older adults but less is known about potential region-specific interactions [19].

Hypertension stands out as the most significant chronic condition and strongest risk factor for greater WMH volume [20]. Strong evidence suggests that cardiovascular factors beyond hypertension, such as cardiac, vascular, or metabolic conditions, have a notable association with WMH volume [21]. Other chronic medical conditions such as kidney disorders [22], respiratory disorders [23], or sleep disorders [24] may also contribute to elevated WMH volume, even after statistically controlling for hypertension. Additionally, in the elderly population, hypertension frequently coexists with other health conditions, which probably exacerbates its adverse effects on white matter [25]. However, the precise relationships between these conditions and WMH across distinct spatial regions are unclear.

The aim of this study was to provide further evidence supporting the presence of distinct spatial patterns of WMHs and to explore how they are linked to comorbidities, AD risk factors, and cognitive performance. To this end, we replicated previously identified WMH patterns found using the recently proposed bullseye representation of WMHs through a data-driven approach [9, 26]. Next, we examined whether comorbidities, sex, Aβ burden, and APOE4 status predict the identified WMH patterns and whether these patterns independently and in interaction with concomitant Aβ pathology are linked to decreased cognitive performance. Finally, we examined whether the number of comorbidities influences the regional WMH burden in the presence or absence of hypertension.

## Methods

### Study population

Data were obtained from the IDcog study, which is a prospective cohort study at the University of Zurich, Switzerland that aims to investigate factors associated with long-term cognitive performance and healthy aging [27]. A total of 233 residents of the greater Zurich area aged 50 to 89 years were recruited via newspaper advertisements. For enrollment in the study, participants had to be at least 50 years of age and German-speaking. Participants were excluded if one of the following criteria was met: presence of a clinically significant depression, evidence of cognitive impairment mainly attributed to another medical condition (e.g., substance abuse, medication, severe heart insufficiency, stroke, cerebrovascular disease, and hepatic encephalopathy), diseases that interfere with current or future study procedures (e.g., severe hearing loss and severe illness), pregnancy, participation in a clinical trial with a substance that may interfere with variables assessed within the present study, or the presence of factors that may interfere with MRI or PET procedures (e.g., pacemaker, clinically relevant tinnitus, and history of significant exposure to radiation). Participants were identified as cognitively unimpaired (CU) or mildly cognitively impaired (MCI) according to published consensus criteria [28].

During the clinical examination, a comprehensive participant profile was obtained, encompassing a detailed medical and neurological examination, medical history, neuropsychological evaluation, blood markers, MRI, Aβ-PET, and APOE genotyping. Additionally, various lifestyle-related factors were evaluated through questionnaires. Essential demographic information, such as age and gender (self-reported as women or men), was also collected during the clinical visit.

### MRI and PET acquisition

We acquired MR and PET images on a 3 T Signa PET/MR scanner (GE Healthcare, Waukesha, WI). Aβ-PET images were acquired from 90 to 110 min postinjection using approximately 140 MBq [18F]-flutemetamol with 4 frames of 5 min each. A BRAVO 3D T1-weighted MRI sequence (8-channel coil) with voxel size 1 mm in sagittal slice orientation, repetition time (TR) = 8.4 ms, echo time (TE) = 3.2 ms, inversion time (TI) = 450 ms, and flip angle = 12° was acquired in parallel to PET acquisition together with the 3D T2-weighted FLAIR image. A standard 3D CUBE FLAIR sequence with a voxel size of 0.48*0.48*0.6 mm was acquired in the sagittal orientation with TR = 6502 ms, TE = 130 ms, TI = 1966 ms, and flip angle = 90°.

### WMH segmentation and parcellation

WMHs were segmented on FLAIR images using the lesion prediction algorithm as implemented in the LST toolbox version 3.0.0 (www.statistical-modelling.de/lst.html) for SPM. The lesion prediction algorithm created the lesion probability maps, which we binarized to create binary lesion masks. A detailed description of WMH segmentation was given in a previous publication [29].

We used the bullseye parcellation approach to obtain regional WMH volumes [26], using a pipeline composed of FreeSurfer [30], AFNI [31], and FSL [32]. Parcellation was performed in each individual FLAIR space by creating a normalized distance map [9] in which a voxel adjacent to the lateral ventricles had an intensity of 0 and a voxel adjacent to the cortex had an intensity of 1. Masks for the lateral ventricles and cortex were acquired using FreeSurfer's pipeline. The normalized distance map was then divided into four concentric, equidistant layers around the ventricles. We then generated individual brain lobe masks by merging the occipital, temporal, parietal, and frontal lobe regions of interests from the Desikan-Killiany atlas [33] and projecting the labels into the white matter until they reached the ventricles. Finally, the bullseye parcellation was created by masking the concentric layers with the brain lobe masks and a mask encompassing the subcortical grey matter structures including bilateral thalamus and basal ganglia (caudate, putamen, and globus pallidus). The anterior and posterior corpus callosum were considered separately as these regions may be specifically affected by vascular and AD pathological processes, respectively [34, 35]. This resulted in 38 parcels for which the WMH volume was calculated.

### Covariate assessment

Covariates were assessed at clinical visits during the patient anamnesis or through questionnaires. The following covariates were included: physical activity, years of education, smoking status, and alcohol use. Physical activity was based on the Lifetime of Experiences Questionnaire [36] and adapted as previously described [27]. For this study, we used the sum of the scores for “early life” (13–30 years) and “mid-life” (30–65 years) age stages. Participants were classified as "alcohol consumers" if they reported drinking on average ≥ 2 alcoholic beverages per day (men) or ≥ 1 alcoholic beverage per day (women), in accordance with the 2018 Swiss alcohol consumption guidelines [37]. Smoking status comprised never, former, or current smoker.

### Comorbidity assessment

Comorbidities were systematically assessed using the cumulative illness rating scale (CIRS) [38]. This scale rates the burden of chronic medical illness across 14 bodily systems while considering the severity of the condition. Each system is rated on a severity scale, ranging from “none” (rated 0) to “extremely severe” (rated 4). If there are several problems in the same system, only the most severe is rated. For instance, for a patient suffering from a well-controlled angina (rated 2) and terminal heart failure (rated 4), only the higher rated condition would be scored in the CIRS cardiac (e.g., rating is 4) [38]. We focused our analysis on scores that could be regarded as potential contributors to WMH. The incorporated scores referred to the following bodily systems in our investigation: cardiac (considers only heart and coronary artery disease), endocrine-metabolic (includes, for instance, diabetes, obesity, and dyslipidemia), hypertension (considers only hypertension), renal (considers only kidney disorders), respiratory (includes, for instance, asthma, pneumonia, and smoking status), and vascular (includes, for instance, vessel, hematopoietic, and lymphopoietic diseases). Due to the relatively low prevalence of scores higher than 1, we dichotomized CIRS scores into 0 (absence of comorbidity) and ≥ 1 (presence of comorbidity). Sleep disorders were defined as present if participants reported experiencing sleep problems other than apnea, sleep apnea, or both.

### AD risk factors

AD risk factors included the presence or absence of at least one APOE4 allele and global Aβ-PET burden. We investigated two previously established Centiloid (CL) cutoff values for Aβ abnormality that mark two relevant inflection points denoting distinct stages of Aβ pathology [39]: a CL of 12 that marks the transition from the absence of pathology to subtle pathology, and a CL of 30 that marks the presence of established pathology.

### Cognitive measures

Participants conducted an extensive neuropsychological examination. In the present study, we focused on three cognitive domains. We calculated composite scores for executive functions (category fluency (animal), letter fluency (s), and figural fluency (5-point test), Stroop’s Test (trial 3), Trail Making Test [B/A]) and episodic memory (CERAD words [learning, recall, and recognition], and VLMT (German version of the RAVLT) [learning, late recall, and recognition], CERAD figures [recall]) as described in our previous work [27]. Additionally, we used the Stroop test (trial 1) and Trail Making Test [A] as measures of processing speed. We converted each individual test score to z-scores using the mean and standard deviation of the cohort and then averaged the z-scores to create the three domain scores.

### Statistical analysis

Cohort characteristics between CU and MCI participants were compared using the Wilcoxon test for continuous variables and the χ^2^ test for categorical variables.

Our first objective was to identify clusters that underlie the distribution of WMHs within the bullseye representation using principal component analysis (PCA). As we aimed to replicate the three components identified in a prior study conducted by Jiménez-Balado et al., we followed their described procedures [9]. The following steps were conducted: (1) adding a constant of 0.01 to all values to avoid log-transformation of zero values, (2) log-transformation of WMH volume for each parcel, (3) standardization of the log-transformed values, and (4) applying PCA followed by an oblimin rotation that allows the extracted components to be correlated. Oblimin-rotated PCA was conducted using the *psych* (v2.3.3) package. Following previous work [9], we assumed three components but also considered two and four components as possible solutions. The final choice regarding the number of selected components was made by evaluating how well these components aligned with those previously identified and by assessing their practical interpretability. The extracted component scores were used in all subsequent analyses.

To investigate the potential associations between component scores and AD-related factors and comorbidities, we employed three linear regression models for all comparisons. These models consisted of (1) an unadjusted model, evaluating the direct association between predictors and component scores; (2) an age-adjusted model, which incorporated age as the sole covariate; and (3) a fully adjusted model, encompassing the covariates age, presence of hypertension (CIRS hypertension), physical activity, education, current smoking status, former smoking status, and alcohol use. This analysis was conducted separately for cognitively unimpaired and cognitively impaired individuals, but it was also performed for the entire cohort. This stratification was done because several risk factors were found to be more prevalent among MCI participants (see Table 1).

**Table 1:** Study cohort characteristics

Characteristics	Total, *n* = 222	CU, *n* = 171 (77%)	MCI, *n* = 51 (23%)
Age, years, mean (SD) [range]	66.2 (8.1) [50 – 89]	64.8 (7.5) [50 – 89]	71 (8.4) [53 – 89]^***^
Women, n (%)	104 (46.8)	87 (50.9)	17 (33.3)^*^
MMSE, mean (SD)	29.2 (1.1)	29.4 (1.0)	28.5 (1.4)^***^
APOE-ε4 carriers, n (%)	53 (23.9)	44 (25.7)	9 (17.6)
WMH volume, ml, median [range]	2.4 [0.1 – 40.2]	2.1 [0.1 – 29.2]	4.2 [0.5 – 40.3]^***^
Antihypertensive medication use	21 (9.5)	14 (8.2)	7 (13.7)
**Aβ burden**			
Centiloids, median [range]	8.3 [-11.8 – 119.5]	8.2 [-11 – 98]	8.4 [-7.9 – 119.5]
CL > 12^a^, n (%)	71 (32.0)	50 (29.2)	21 (41.1)
CL > 30, n (%)	24 (10.8)	12 (7.0)	12 (23.5)^**^
**Cognition, z-scores mean (SD)**			
Episodic memory, (N/A = 3)	0.02 (0.8)	0.3 (0.52)	-0.99 (0.82)^***^
Executive functions, (N/A = 4)	0.02 (0.63)	0.14 (0.57)	-0.41 (0.68)^***^
Processing speed, (N/A = 1)	0 (0.8)	0.1 (0.77)	-0.34 (0.81)^***^
**Comorbidities (CIRS rating > 0), n (%)**			
Hypertension	75 (33.7)	51 (29.8)	24 (47.1)^*^
Cardiac	48 (21.6)	35 (20.5)	13 (25.5)
Vascular	45 (20.3)	33 (19.3)	12 (23.5)
Renal	30 (13.5)	18 (10.5)	12 (23.5)^*^
Endocrine-metabolic	155 (70.0)	122 (71.3)	33 (64.7)
Respiratory	62 (28.0)	46 (26.9)	16 (31.4)
Sleep disorder, n (%)	64 (28.8)	51 (29.8)	13 (25.5)
**Lifestyle Factors, mean (SD) *or* n (%)**			
Early/mid-life physical activity (N/A = 11)	25.3 (17)	26.3 (17.9)	21.9 (13.0)
Education, years, mean (SD)	15.4 (2.8)	15.6 (2.8)	14.9 (2.9)
Current smoker	35 (15.8)	32 (18.7)	3 (5.9)^*^
Former smoker	79 (36.6)	51 (29.8)	28 (54.9)^**^
Alcohol use (N/A = 10)	44 (20.8)	33 (20.2)	11 (22.4)

Statistical differences in the characteristics between the CU and MCI groups are highlighted, with significance levels denoted as *p < 0.05, **p < 0.01, and ***p < 0.001

*Abbreviations*: *SD* standard deviation, *N/A* not available

^a^Including participants with CL > 30

We employed multiple linear regression models to investigate the impact of the components on cognitive domain composite scores, while accounting for Aβ status (CL > 12), education, age, and sex as covariates. In a subsequent analysis, we introduced an interaction term, Aβ status * component score, into all models. To mitigate the influence of potential outliers, each model was repeated using robust linear regression, and we designated an effect as significant if the robust confidence interval did not include 0.

Finally, we explored the relationships between the number of comorbidities and component scores, considering potential interactions with hypertension, the factor most strongly associated with increased WMH volume [20]. Due to the relatively small sample sizes in some subgroups, we examined associations using graphical representations of the data but also reported significant group differences using the Wilcoxon test.

Analyses were performed in R version 4.2.2. A two-sided *p* value < 0.05 or a 95% confidence interval not including 0 was considered statistically significant. Given the high correlations observed among both the WMH components and the comorbidities, we did not apply correction for multiple comparisons. However, we have provided 95% confidence intervals for all our findings to assess the uncertainty of each association.

## Results

The total IDcog cohort consists of 233 participants, of whom 11 were excluded due to artifacts on the FLAIR image (*n* = 8), missing FLAIR image (*n* = 1), or bullseye parcellation outlining issues due to a large postischemic lesion (*n* = 2). Thus, our final cohort comprised 222 participants, with 171 (77%) classified as cognitively unimpaired and 51 (23%) diagnosed with MCI. The characteristics of the study cohort are shown in Table 1.

### Identification of WMH principal components

**Fig. 1:**
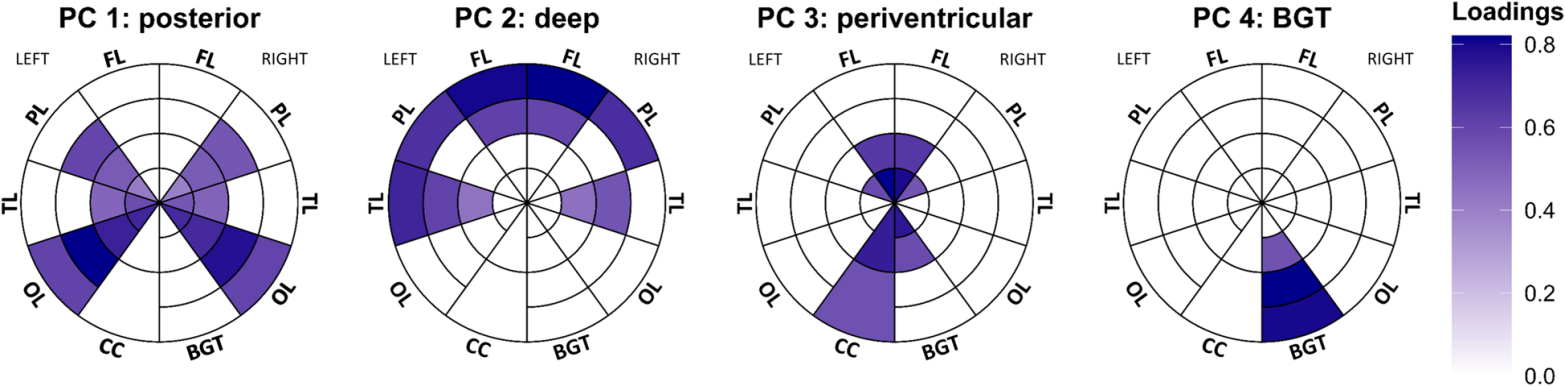
Principal component analysis identified four patterns of hyperintensity signal distribution. Plots show the loading in each parcel for each component in the bullseye representation. The concentric rings in the Bullseye diagram delineate the area between the ventricular surface and the cortex into four equidistant layers. The interior parcels in the plot represent the most periventricular area, and the most external parcels correspond to the regions below the cortex. The corpus callosum (CC) was divided into anterior (inner parcel) and posterior (external parcel) regions. Only loadings > 0.4 are shown in the plots. Abbreviations: BGT = basal ganglia & thalamus; CC = corpus callosum; FL = frontal lobe; OL = occipital lobe; PL = parietal lobe; TL = temporal lobe

**Fig. 2:**
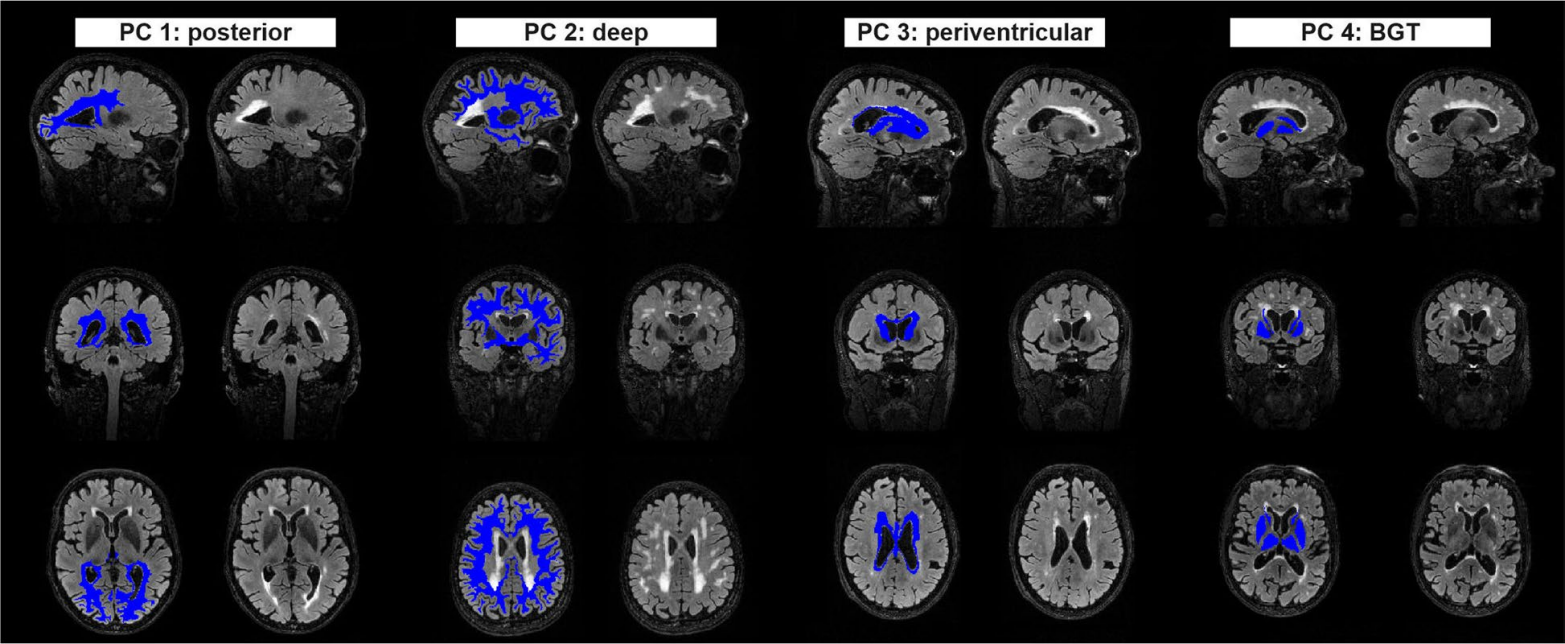
FLAIR images of participants exhibiting the highest principal component scores for each component, with and without overlaid bullseye parcels. Only parcels with loadings > 0.4 are indicated in the figure

Using PCA, we identified four WMH components. These components are depicted in the bullseye representation in Fig. 1 and overlaid on the respective participant’s FLAIR image with the highest component score in Fig. 2. The first component largely explained the variance in WMHs mainly located in the occipital and periventricular parietal regions (named the posterior WMH component). The second component largely explained the variance in the deep frontal, parietal, and temporal regions (deep WMH component). The third component explained variance in periventricular regions (periventricular WMH component). These three components closely match previously identified components by Jiménez-Balado et al. [9] and account for 25% (posterior WMH component), 20% (deep), and 18% (periventricular) of the total WMH distribution variance. The fourth component corresponded to hyperintensities within the bilateral basal ganglia and thalamus mask (BGT component). Although this component accounted for a relatively small proportion of the total hyperintensity signal distribution (8%), it is still considered significant because it indicates signals in and around specific subcortical gray matter structures. We therefore selected the four-component solution rather than the three-component alternative. Older age was similarly associated with increased posterior (r_Pearson_ = 0.57, *p* < 0.001), deep (r_Pearson_ = 0.40, *p* < 0.001), and periventricular (r_Pearson_ = 0.52, *p* < 0.001) component scores but showed a weaker association with the BGT component (r_Pearson_ = 0.15, *p* = 0.02). Similarly, global WMH volume showed high correlations with posterior (r_Pearson_ = 0.84), deep (r_Pearson_ = 0.73), and periventricular (r_Pearson_ = 0.85) WMH components but showed a weaker association with the BGT component (r_Pearson_ = 0.32), although all associations were strong (all *p* < 0.001). After adjusting for age and the presence of hypertension, no difference in component scores was found between CU and MCI participants (*p* > 0.08).

### Associations with comorbidity and AD risk factors

**Fig. 3:**
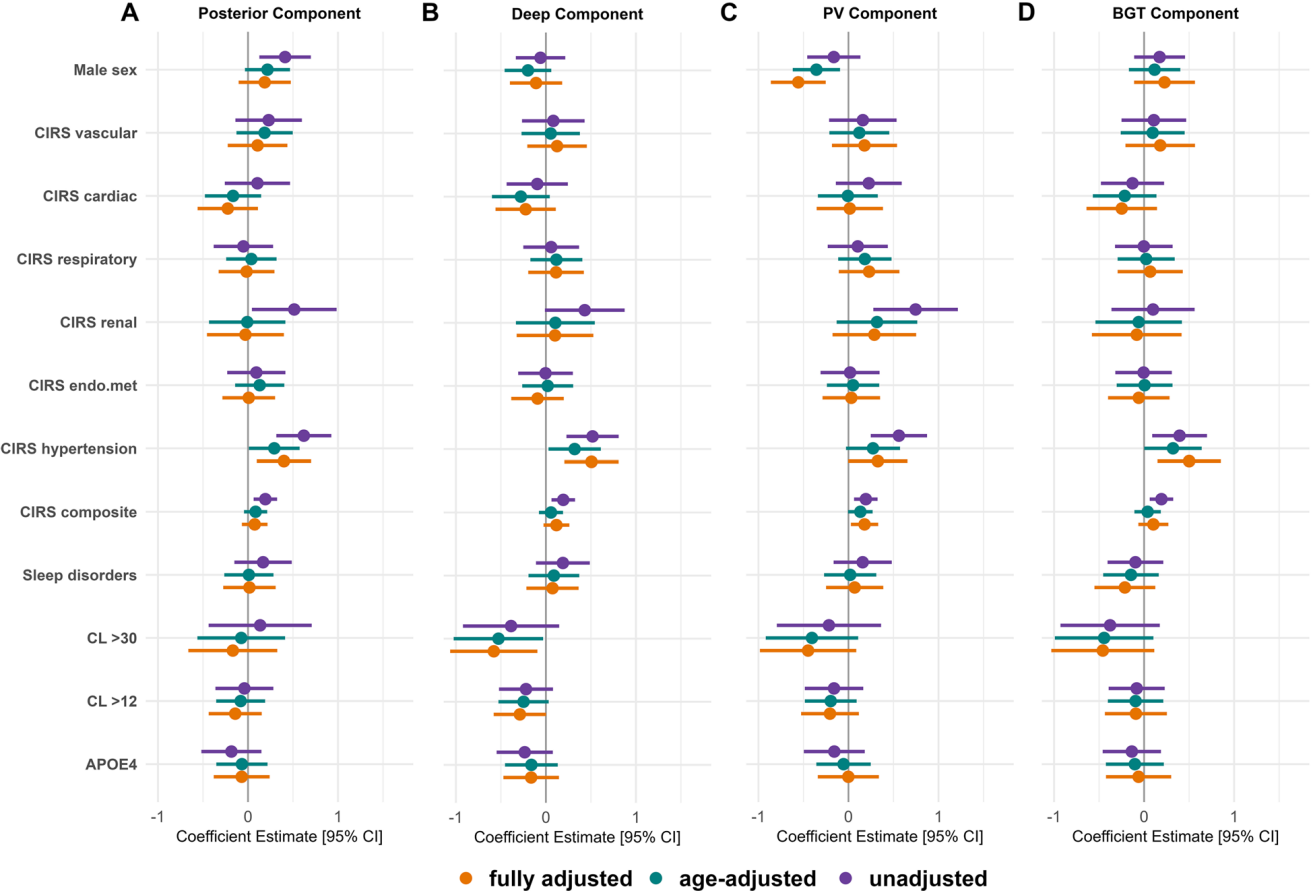
Coefficient estimates for each risk factor across principal components in the CU sample. Fully adjusted models included the covariates age, presence of hypertension (CIRS hypertension), physical activity, education, current smoking status, former smoking status, and alcohol use. Sex was coded as female = 0 and male = 1; thus, negative coefficient estimates indicate higher component scores in female compared to male individuals

**Fig. 4:**
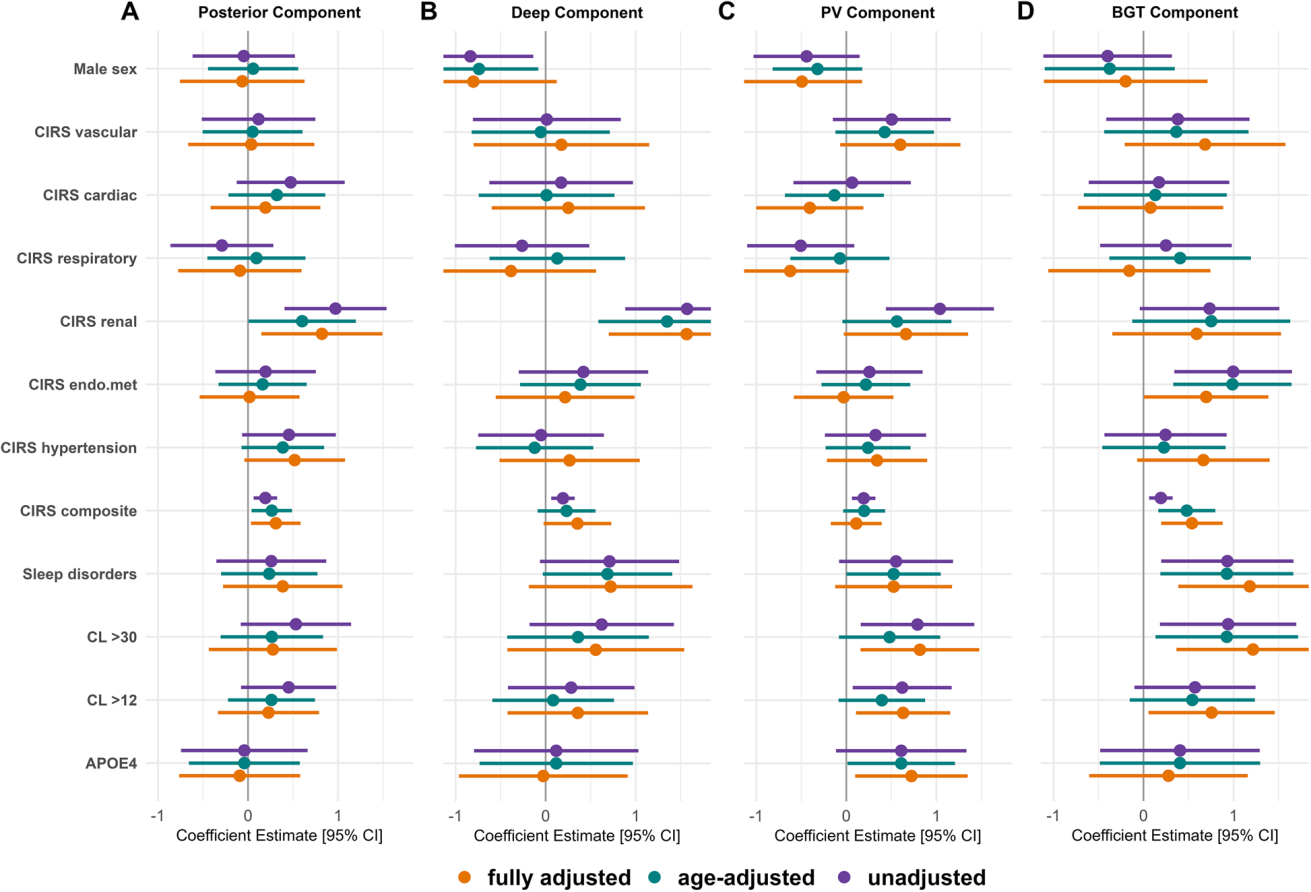
Coefficient estimates for each risk factor across principal components in the MCI sample. Fully adjusted models included the covariates age, presence of hypertension (CIRS hypertension), physical activity, education, current smoking status, former smoking status, and alcohol use. Sex was coded as female = 0 and male = 1; thus, negative coefficient estimates indicate higher component scores in female compared to male individuals

The associations of component scores with the risk factors studied are summarized in Figs. 3 and 4. The results of the analysis using the combined sample (CU + MCI) are available in Additional file 1 (Supplementary Fig. 1). Complete model estimates, confidence intervals, and *p*-values are also available in Additional file 1 (Supplementary Table 1-3). In the fully adjusted models, among the covariates, higher education was associated with lower deep (β = -0.21, *P* = 0.002) and BGT (β = -0.19, *P* = 0.008) WMH component scores, and higher physical activity was associated with lower posterior (β = -12, *P* = 0.034) WMH component scores (Supplementary Tables 4, 5 and 6). Hypertension and CIRS renal generally exhibited the most robust associations with elevated WMH component scores. However, hypertension appeared to have a stronger connection with higher component scores in CU participants, while CIRS renal tended to be more closely linked to higher component scores in individuals in the MCI stage. Female sex was associated with higher periventricular component scores, particularly in CU participants. Among the AD-related factors, in CU participants, having elevated Aβ burden was associated with lower scores in the deep WMH component. In participants with MCI, both having CL > 12 and having CL > 30 were linked to higher scores in the periventricular and BGT components. The association between APOE4 and the periventricular component lost significance when Aβ status (CL > 12) was accounted for in the model, indicating that Aβ load is the decisive factor for this association.

### Associations with cognition

**Fig. 5:**
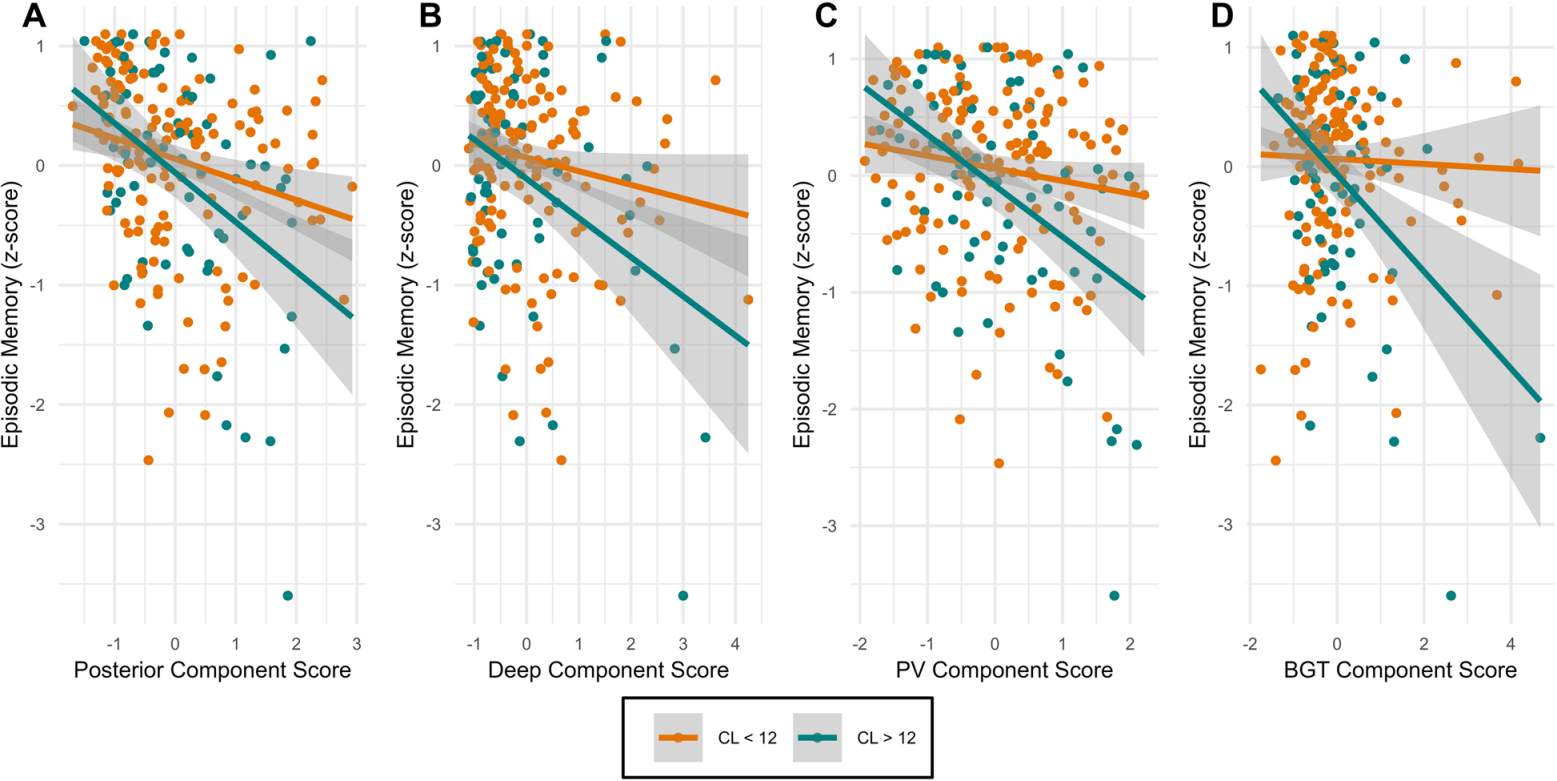
Principal component scores are associated with lower episode memory performance in individuals with elevated Aβ burden

Adjusting for covariates, none of the components were associated with cognitive performance in any of the three investigated cognitive domains (Table 2). Of the covariates, younger age, more years of education, and female sex were associated with better cognitive performance, but the main effect of Aβ status (CL > 12) was not. When a component score*Aβ status interaction term was included in the models, we observed interactions between Aβ status and all components on episodic memory performance (Fig. 5). The only exception was the deep component, where the robust confidence interval did encompass 0. Compared to the model without an interaction term, incorporating a component*Aβ interaction improved the model fit, as shown by a significant likelihood ratio test (posterior: *p* = 0.001; periventricular: *p* < 0.001; BGT: *p* < 0.001) and resulted in an increase in the adjusted R^2^ (posterior: ΔR^2^ = 0.032; periventricular: ΔR^2^ = 0.042; BGT: ΔR^2^ = 0.042). Jointly incorporating Aβ interactions with the periventricular and BGT components in the model revealed significant associations with memory performance for both interactions (periventricular*Aβ status: β = -0.27, *p* = 0.01; BGT*Aβ status: β = -0.28, *p* = 0.011). When Aβ status interactions with the periventricular and posterior components were included simultaneously, only the interaction with the periventricular component remained significant (periventricular*Aβ status: β = -0.29, *p* = 0.023; posterior*Aβ status: β = -0.14, *p* = 0.259). The results did not change when we additionally controlled for the absence or presence of hypertension.

**Table 2:** Regression coefficients of WMH components and their interaction with Aβ status on cognitive domains

	Posterior Component Score			Posterior Component Score*Aβ		
**Cognitive domain**	**β (95% CI)**	***p*-value**	**Robust 95% CI**	**β (95% CI)**	***p*-value**	**Robust 95% CI**
Episodic Memory	-0.02 (-0.13 to 0.09)	0.17	-0.12 to 0.10	-0.34 (-0.54 to -0.13)	0.001	-0.48 to -0.10
Executive Function	-0.05 (-0.14 to 0.05)	0.39	-0.14 to 0.05	-0.10 (-0.27 to 0.07)	0.27	-0.28 to 0.07
Processing Speed	0.03 (-0.09 to 0.14)	0.67	-0.08 to 0.16	-0.01 (-0.22 to 0.20)	0.89	-0.22 to 0.20
	Deep Component Score			Deep Component Score*Aβ		
**Cognitive domain**	**β (95% CI)**	***p*-value**	**Robust 95% CI**	**β (95% CI)**	***p*-value**	**Robust 95% CI**
Episodic Memory	-0.05 (-0.15 to 0.051)	0.32	-0.14 to 0.08	-0.24 (-0.43 to -0.05)	0.013	-0.35 to 0.02
Executive Function	0.04 (-0.05 to 0.12)	0.40	-0.4 to 0.21	-0.07 (-0.24 to 0.09)	0.38	-0.25 to 0.08
Processing Speed	-0.01 (-0.12 to 0.09)	0.73	-0.13 to 0.08	-0.02 (-0.21 to 0.18)	0.87	-0.19 to 0.21
	Periventricular Component Score			Periventricular Component Score*Aβ		
**Cognitive domain**	**β (95% CI)**	***p*-value**	**Robust 95% CI**	**β (95% CI)**	***p*-value**	**Robust 95% CI**
Episodic Memory	-0.09 (-0.20 to 0.02)	0.11	-0.17 to 0.03	-0.37 (-0.57 to -0.18)	< 0.001	-0.51 to -0.14
Executive Function	-0.05 (-0.14 to 0.04)	0.17	-0.12 to 0.07	-0.07 (-0.24 to 0.10)	0.41	-0.27 to 0.08
Processing Speed	0.07 (-0.04 to 0.19)	0.20	-0.05 to 0.28	-0.08 (-0.28 to 0.12)	0.44	-0.26 to 0.14
	BGT Component Score			BGT Component Score*Aβ		
**Cognitive domain**	**β (95% CI)**	***p*-value**	**Robust 95% CI**	**β (95% CI)**	***p*-value**	**Robust 95% CI**
Episodic Memory	-0.05 (-0.14 to 0.05)	0.43	-0.09 to 0.09	-0.38 (-0.58 to -0.18)	< 0.001	-0.53 to -0.12
Executive Function	0.07 (-0.01 to 0.15)	0.09	-0.01 to 0.15	-0.02 (-0.20 to 0.15)	0.82	-0.21 to 0.16
Processing Speed	-0.03 (-0.13 to 0.07)	0.33	-0.15 to 0.04	-0.06 (-0.27 to 0.15)	0.57	-0.33 to 0.09

For Aβ status, a binarized variable with a Centiloid cutoff of 12 was used. Participants who did not complete all neuropsychological tests included in the composite scores for episodic memory (*n* = 3), executive function (*n* = 4), and processing speed (*n* = 1) were excluded from this analysis

### Impact of hypertension on component scores in the presence of comorbidities

**Fig. 6:**
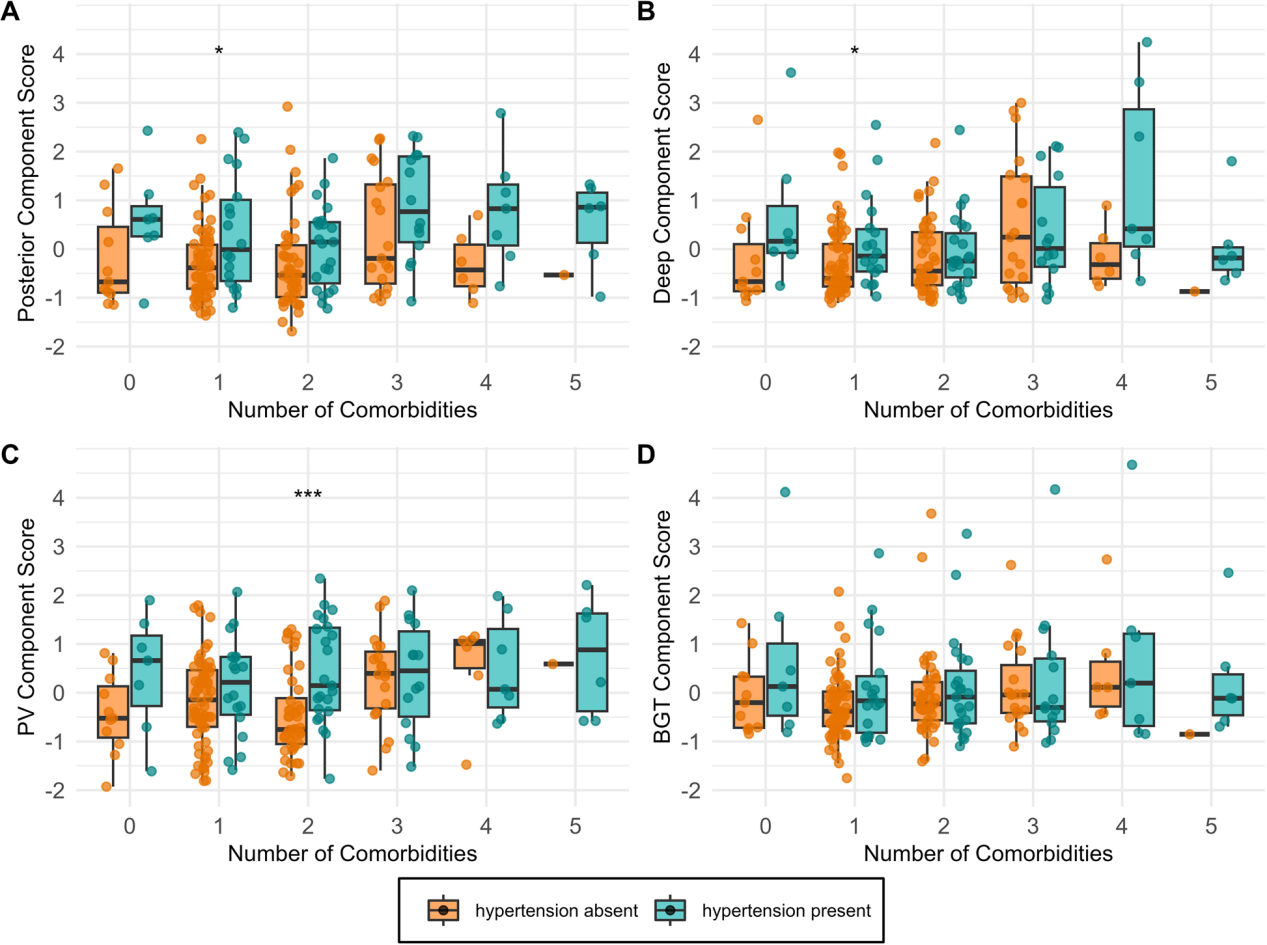
Hypertension was the primary driver of elevated WMH component scores, irrespective of the number of comorbidities. Comorbidities included CIRS scores of vascular, cardiac, respiratory, renal, and endocrine-metabolic systems and sleep disorders

Visual assessment of the relationship between the number of comorbidities, with or without hypertension, and component scores suggests that the presence of hypertension is associated with higher component scores, regardless of the number of comorbidities (Fig. 6). In cases where hypertension is absent, having three or more comorbidities appears to lead to a similar WMH burden. This observation is most pronounced in the periventricular component (Fig. 6C) but less so in the BGT component. However, statistically significant differences in the components between participants with and without hypertension are generally not observed for most comparisons.

## Discussion

Using principal component analysis, we identified three spatial WMH patterns that describe variance in posterior, periventricular, and deep white matter regions. A fourth component selectively described signal hyperintensities in and around subcortical grey matter structures (BGT component). In our CU sample, the presence of hypertension emerged as the primary predictor of elevated component scores across all WMH patterns, except for the periventricular component. Our results in the MCI group indicated a more intricate relationship between risk factors and WMH patterns. Specifically, we observed patterns of risk factors associated with one component (e.g., AD-related factors with periventricular component), single factors associated with multiple components (e.g., CIRS renal with posterior and deep component), and certain factors that were selectively associated with only one component (e.g., sleep disorders with BGT component). Furthermore, in the combined sample (CU + MCI), we found that higher component scores, especially in the periventricular and BGT components, predicted poorer episodic memory when abnormal Aβ levels were present (Centiloid > 12), whereas the main effects of the WMH components and Aβ status did not. Finally, our results underscore the importance of hypertension as a major factor in increasing WMH volume, even in the absence of comorbidities.

The identified WMH components in our work closely match previously identified components [9]. Replication of these WMH components in a separate cohort using a different lesion segmentation tool underscores the credibility of the components identified using the present approach. Similarities with two previous studies that used data-driven approaches to define spatial WMH patterns are also apparent [5, 8]. In all studies, patterns that separate deep from periventricular WMHs and posterior from anterior WMHs are observed. However, these studies also identified further subdivisions within the patterns found in our work, dividing periventricular WMHs further into frontal and dorsal clusters [8] or posterior WMHs into parietal and occipital clusters [5]. Additionally, one study identified a juxtacortical WMH cluster [5]. Differences in the identified clusters may be attributed to variations in the study cohorts, including factors such as age, age range, and level of cognitive impairment. Another potential explanation relates to methodological differences. Unlike previous studies that utilized clustering methods restricting cluster overlap [5] or maximizing cluster distinctness [8], our approach allows for correlated components. Nonoverlapping clusters offer the advantage of clearer separation of cluster-specific risk factors. On the other hand, overlapping clusters may better reflect reality, considering that certain risk factors, such as age and hypertension, exert some degree of influence on WMH burden across all regions. Various factors, including differences in arterial supply and regional vulnerability to underlying disease etiologies, may account for the emergence of unique WMH patterns [40]. Alternatively, some patterns may reflect different disease stages. For instance, more periventricular WMH localization could reflect an earlier stage of disease in many individuals given that lesions are often observed first in periventricular regions [41].

We also identified a component characterized by hyperintense signals in the basal ganglia and thalamus, a pattern aligning with a previous study that used predefined WMH patterns as a reference [42] but distinct from all patterns reported in the data-driven studies mentioned earlier. This component likely includes signals around subcortical gray matter structures and may also reflect hyperintensity within these structures, which therefore cannot be classified as WMH [43]. Thus, we discuss this component separately from the others. Unlike the other components, which displayed strong intercorrelations and robust associations with age and the total WMH volume, the BGT component exhibited weaker correlations with the other three components, showed a less robust relationship with age, and demonstrated a less pronounced correlation with the total WMH volume. Notably, despite accounting for only 8% of the total hyperintensity signal variance, hyperintensities within these regions may still be of clinical importance, as they showed significant associations with episodic memory performance in the presence of increased Aβ burden. This association appears to be partially independent of the Aβ*WMH interaction observed in other regions, suggesting distinct mechanisms contributing to memory decline. Our finding of a link between hypertension and higher BGT component scores aligns with existing research highlighting the vulnerability of these structures to the detrimental effect of chronically elevated blood pressure [44]. The connections between Aβ burden, sleep disorders, and endocrine/metabolic conditions with the BGT component are interesting findings that could provide clues about the potential consequences or mechanisms driving hyperintensity development in these regions.

In the CU sample, we found that hypertension was the only condition significantly associated with higher component scores. This was the case for all components except the periventricular component, for which the association no longer reached significance after adjusting for age and other confounders. That these associations appear to be most robust in the deep and BGT patterns is consistent with the literature [5, 42]. Both regions are particularly vulnerable to vascular insufficiency [44]. The visual examination of Fig. 6 indicates that there is no apparent interaction between hypertension and other comorbidities concerning regional WMHs. Notably, the mere presence of hypertension, even in the absence of other comorbid conditions, appears sufficient to elevate WMH volume. Among individuals with a low number of comorbid diseases but without hypertension, component scores do not reach the levels observed in individuals with hypertension. Only when multiple comorbidities are present do the scores increase and approximate those of individuals with hypertension. In summary, these findings highlight hypertension as the primary factor influencing regional WMH volume, and this influence appears to be largely unaffected by other comorbidities.

Female sex was associated with increased periventricular component scores in our study. This finding aligns with previous research showing higher total WMH volume in females than in males [13, 14]. However, our findings differ from other studies that have investigated regional WMH volume. These previous studies primarily reported higher WMH burden in deep brain regions among females compared to males [10, 15], a finding that we observed to some degree in the MCI sample but not in the CU sample. One potential explanation for this difference is that the WMH burden in deep white matter regions in our CU sample may have been low, making differences harder to detect. In the MCI sample, we also found a strong association between CIRS renal and higher deep component scores. This association remained significant even after accounting for age, hypertension, and other covariates, suggesting that there are other mechanisms beyond a shared susceptibility of the kidneys and the brain to hypertensive conditions [45], that contribute to increased WMH volume in deep white matter regions in individuals with renal disorders [46]. Increased Aβ burden and the presence of an APOE4 allele were associated with elevated periventricular component scores in the MCI sample. However, the association with APOE4 disappeared when accounting for Aβ burden, suggesting that APOE4's effect was primarily due to its correlation with higher Aβ burden. Although a periventricular WMH distribution has been observed in AD patients [18], previous studies reported a stronger correlation between a posterior WMH distribution and Aβ pathology [5]. While our analysis combining the CU and MCI samples provided some evidence for this association, the association between the posterior component and Aβ abnormality became nonsignificant after age adjustment, suggesting that this link may be weaker in earlier Aβ stages and possibly downstream of other common age-related factors.

Our findings indicate that higher WMH scores were associated with worse cognition only in individuals with increased Aβ burden. The interaction between Aβ abnormality and WMHs was most robustly observed for the periventricular and BGT components and was specific for episodic memory performance. This observation helps clarify why in the CU sample, Aβ abnormality was associated with lower WMH component scores. Individuals with elevated Aβ burden need to have a lower WMH burden to remain cognitively healthy. When both pathologies are elevated within an individual, cognitive decline is accelerated [19], increasing the likelihood of developing mild cognitive impairment. This interpretation is further supported by the positive association of Aβ abnormality with periventricular component scores in the MCI sample. One explanation for the selective association with episodic memory may be that the memory assessment used was more sensitive in detecting impairment than the other cognitive measures. Alternatively, WMHs might directly compromise memory systems through brain atrophy [47]. Multiple studies have observed that medial temporal lobe atrophy mediates the effect of WMH volume on memory performance [48, 49]. The association between WMHs and medial temporal atrophy appears to be at least partially independent of Aβ-associated atrophy in these regions [50]. In turn, periventricular WMHs may disrupt crucial long-range fiber tracts [51], which might explain the strong interaction between Aβ status and the periventricular WMH component on episodic memory. Alternatively, as periventricular WMHs tend to be the earliest manifestation of WMHs and are strongly correlated with global WMH volume [8, 52], the total WMH burden may be the decisive factor in a healthy cohort.

## Limitations

An important aspect to consider when interpreting our results is our study population, which was a relatively young and healthy cohort of volunteers who were motivated to participate in this cohort study. This is reflected in the CIRS scores, which indicate that most participants were free of mild or severe comorbidities. Thus, our ability to detect associations of WMH components with risk factors or cognitive domains may have been limited. Additionally, due to the limited number of participants with more than three comorbid conditions, our interpretation of the last analysis relied on visual assessment of the figure and should be viewed with caution. Furthermore, we found that age has a strong effect on all components except for the BGT component, which may complicate the accurate quantification of the effects of other age-related risk factors, such as hypertension. Future studies might consider dividing the cohorts into subgroups of similar age or examine how factors such as hypertension moderate the effect of age on regional WMH patterns. Finally, we note that the identified components, except for the BGT component, are highly correlated with one another and with the total WMH volume, which currently limits their usefulness as clinically relevant biomarkers. Clustering approaches that consider not only the location but also the morphology of WMHs (e.g., punctate vs. confluent WMHs) and concomitant pathologies are desirable. Investigating the microstructural heterogeneity of WMH using diffusion MRI may also provide further insight into understanding their clinical and pathologic correlates and underlying pathologic features [53].

## Conclusion

Our findings contribute to the growing understanding that WMHs are not solely attributed to a heterogeneous constellation of risk factors but may have both shared and region-specific underlying etiologies. Furthermore, our findings suggest location-specific interactions between WMH and AD pathology on cognition in early disease stages, as the co-occurrence of relatively low levels of Aβ pathology and WMHs, particularly in periventricular regions and deep gray matter structures, was associated with poorer episodic memory performance.

### Supplementary Information

**Supplementary Material 1. **

## Data Availability

The datasets used and analysed during the current study are available from the corresponding author on reasonable request after evaluation by the authors and, if applicable, by the local ethics authority.

## Abbreviations

AD: Alzheimer’s disease
Aβ: Amyloid-β
APOE4: Apolipoprotein E4
BGT: Basal ganglia & thalamus
CL: Centiloid
CIRS: Cumulative illness rating scale
CU: Cognitively unimpaired
FLAIR: Fluid attenuated inversion recovery
MCI: Mild cognitive impairment
MRI: Magnetic resonance imaging
PCA: Principal component analysis
PET: Positron emission tomography
PV: Periventricular
WMH: White matter hyperintensity
