# Supplementary material for: White matter hyperintensity patterns: associations with comorbidities, amyloid, and cognition
Source: Alzheimers Res Ther. 2024 Apr 1;16:67. doi: 10.1186/s13195-024-01435-6 (PMC10983708; doi:10.1186/s13195-024-01435-6)
Supplement: Supplementary file 1 — Supplementary Material 1. [file 13195_2024_1435_MOESM1_ESM.docx]

**White matter hyperintensity patterns: associations with comorbidities, amyloid, and cognition**

*– Supplementary Materials –*

**Supplementary Figure 1. Coefficient estimates for each risk factor across** **principal components in the combined sample (CU + MCI).** Fully adjusted models included the covariates age, presence of hypertension (CIRS hypertension), physical activity, education, current smoking status, former smoking status, and alcohol use. Sex was coded as female = 0 and male = 1; thus, negative estimates indicate higher component scores in female compared to male individuals.


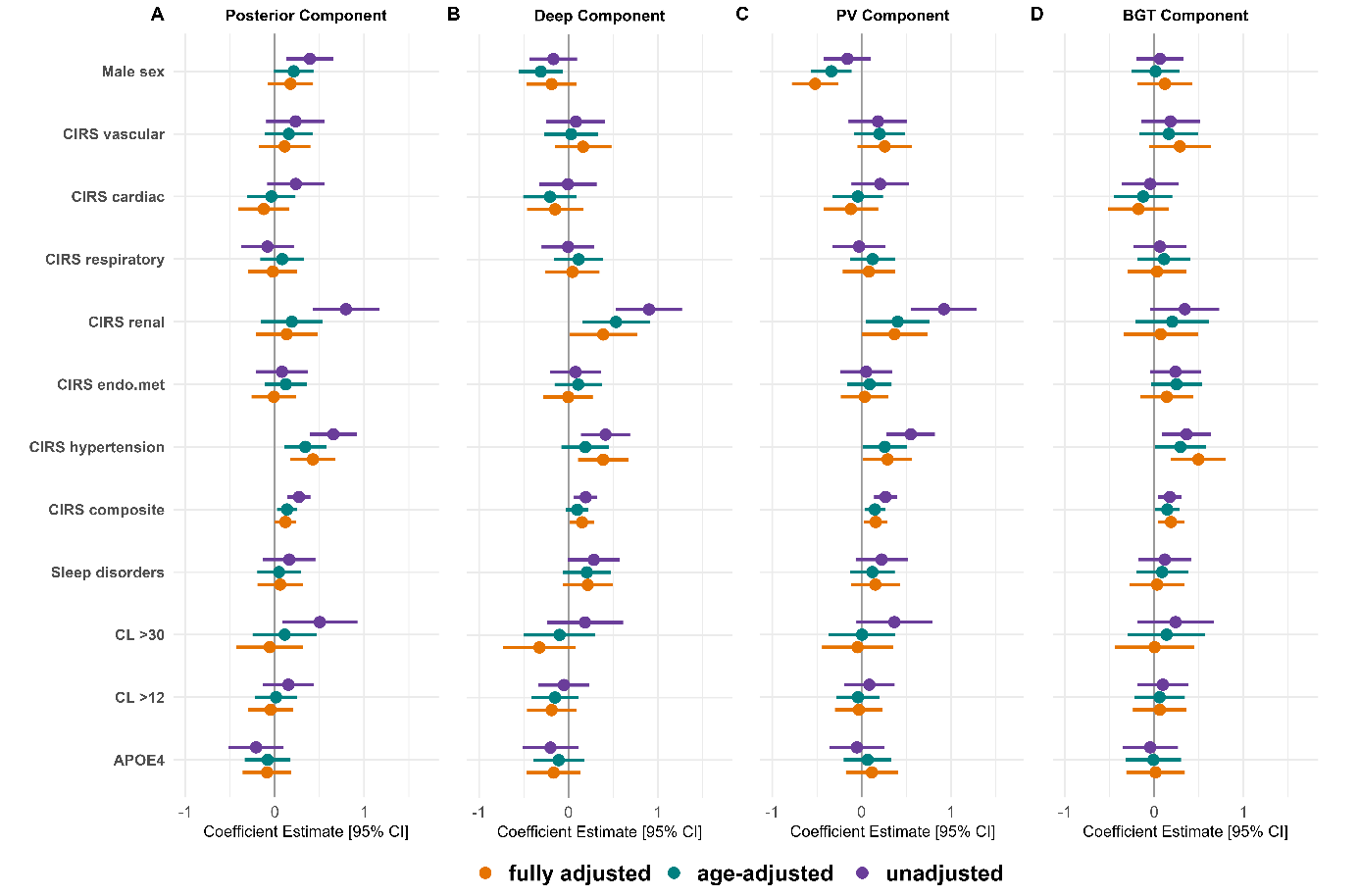


**Supplementary Table 1. Detailed estimates for each risk factor across principal components in the combined sample (CU + MCI) with 95% confidence intervals (CI).** Sex was coded as female = 0 and male = 1; thus, negative estimates indicate higher component scores in female compared to male individuals. Abbreviations: comp = component; post = posterior; BGT = basal ganglia & thalamus; PV = periventricular. **P* < 0.05; ***P* < 0.01; ****P* < 0.001.

|  |  | Unadjusted | | | Age-adjusted | | | Fully adjusted | | |
| --- | --- | --- | --- | --- | --- | --- | --- | --- | --- | --- |
| Comp. | Risk factor | β | CI low | CI high | β | CI low | CI high | β | CI low | CI high |
| Post. | Male sex | **0.39**** | **0.13** | **0.65** | 0.21 | -0.01 | 0.43 | 0.18 | -0.08 | 0.43 |
| Post. | CIRS vascular | 0.23 | -0.10 | 0.56 | 0.16 | -0.11 | 0.43 | 0.11 | -0.18 | 0.40 |
| Post. | CIRS cardiac | 0.24 | -0.09 | 0.56 | -0.04 | -0.31 | 0.23 | -0.12 | -0.41 | 0.16 |
| Post. | CIRS respiratory | -0.08 | -0.38 | 0.21 | 0.08 | -0.16 | 0.33 | -0.02 | -0.30 | 0.25 |
| Post. | CIRS renal | **0.80***** | **0.42** | **1.17** | 0.19 | -0.15 | 0.54 | 0.13 | -0.21 | 0.48 |
| Post. | CIRS endo.met | 0.08 | -0.21 | 0.37 | 0.12 | -0.11 | 0.36 | -0.01 | -0.26 | 0.24 |
| Post. | CIRS hypertension | **0.66***** | **0.39** | **0.92** | **0.34**** | **0.10** | **0.58** | **0.43**** | **0.17** | **0.68** |
| Post. | CIRS composite | **0.27***** | **0.14** | **0.40** | **0.14*** | **0.02** | **0.25** | 0.12 | -0.01 | 0.24 |
| Post. | Sleep disorders | 0.16 | -0.13 | 0.45 | 0.05 | -0.20 | 0.29 | 0.06 | -0.19 | 0.32 |
| Post. | CL >30 | **0.50*** | **0.08** | **0.92** | 0.11 | -0.25 | 0.47 | -0.06 | -0.43 | 0.31 |
| Post. | CL >12 | 0.15 | -0.13 | 0.44 | 0.01 | -0.22 | 0.25 | -0.05 | -0.30 | 0.20 |
| Post. | APOE4 | -0.21 | -0.52 | 0.10 | -0.08 | -0.34 | 0.18 | -0.09 | -0.36 | 0.19 |
| BGT | Male sex | 0.07 | -0.20 | 0.33 | 0.02 | -0.25 | 0.28 | 0.12 | -0.18 | 0.42 |
| BGT | CIRS vascular | 0.18 | -0.15 | 0.51 | 0.16 | -0.16 | 0.49 | 0.29 | -0.06 | 0.63 |
| BGT | CIRS cardiac | -0.05 | -0.37 | 0.28 | -0.12 | -0.45 | 0.20 | -0.18 | -0.52 | 0.16 |
| BGT | CIRS respiratory | 0.06 | -0.23 | 0.36 | 0.11 | -0.19 | 0.40 | 0.03 | -0.30 | 0.36 |
| BGT | CIRS renal | 0.34 | -0.04 | 0.73 | 0.20 | -0.21 | 0.62 | 0.07 | -0.34 | 0.49 |
| BGT | CIRS endo.met | 0.24 | -0.05 | 0.52 | 0.25 | -0.04 | 0.53 | 0.14 | -0.16 | 0.44 |
| BGT | CIRS hypertension | **0.36*** | **0.09** | **0.64** | **0.29*** | **0.01** | **0.58** | **0.49**** | **0.19** | **0.80** |
| BGT | CIRS composite | **0.18**** | **0.04** | **0.31** | **0.15*** | **0.01** | **0.28** | **0.19*** | **0.04** | **0.34** |
| BGT | Sleep disorders | 0.12 | -0.17 | 0.41 | 0.09 | -0.20 | 0.38 | 0.03 | -0.27 | 0.34 |
| BGT | CL >30 | 0.24 | -0.19 | 0.66 | 0.14 | -0.29 | 0.57 | 0.00 | -0.44 | 0.45 |
| BGT | CL >12 | 0.10 | -0.19 | 0.38 | 0.06 | -0.22 | 0.34 | 0.06 | -0.24 | 0.36 |
| BGT | APOE4 | -0.04 | -0.36 | 0.27 | -0.01 | -0.32 | 0.30 | 0.02 | -0.31 | 0.34 |
| PV | Male sex | -0.16 | -0.42 | 0.11 | **-0.34**** | **-0.56** | **-0.11** | **-0.52***** | **-0.78** | **-0.26** |
| PV | CIRS vascular | 0.18 | -0.15 | 0.51 | 0.20 | -0.08 | 0.48 | 0.26 | -0.05 | 0.57 |
| PV | CIRS cardiac | 0.21 | -0.11 | 0.53 | -0.04 | -0.32 | 0.24 | -0.12 | -0.42 | 0.19 |
| PV | CIRS respiratory | -0.03 | -0.32 | 0.27 | 0.12 | -0.13 | 0.38 | 0.08 | -0.21 | 0.38 |
| PV | CIRS renal | **0.92***** | **0.55** | **1.29** | **0.40*** | **0.05** | **0.76** | **0.37*** | **0.00** | **0.74** |
| PV | CIRS endo.met | 0.05 | -0.24 | 0.34 | 0.09 | -0.16 | 0.34 | 0.03 | -0.23 | 0.30 |
| PV | CIRS hypertension | **0.55***** | **0.28** | **0.82** | **0.26*** | **0.01** | **0.51** | **0.29*** | **0.02** | **0.56** |
| PV | CIRS composite | **0.27***** | **0.14** | **0.40** | **0.15*** | **0.03** | **0.26** | **0.16*** | **0.03** | **0.29** |
| PV | Sleep disorders | 0.22 | -0.07 | 0.52 | 0.12 | -0.13 | 0.37 | 0.15 | -0.12 | 0.43 |
| PV | CL >30 | 0.37 | -0.06 | 0.79 | 0.01 | -0.37 | 0.38 | -0.04 | -0.44 | 0.35 |
| PV | CL >12 | 0.09 | -0.20 | 0.37 | -0.04 | -0.29 | 0.20 | -0.03 | -0.30 | 0.24 |
| PV | APOE4 | -0.05 | -0.36 | 0.26 | 0.07 | -0.20 | 0.34 | 0.12 | -0.18 | 0.41 |
| Deep | Male sex | -0.17 | -0.43 | 0.10 | **-0.31*** | **-0.55** | **-0.06** | -0.19 | -0.47 | 0.09 |
| Deep | CIRS vascular | 0.08 | -0.25 | 0.41 | 0.03 | -0.27 | 0.33 | 0.16 | -0.15 | 0.48 |
| Deep | CIRS cardiac | -0.01 | -0.33 | 0.32 | -0.21 | -0.51 | 0.09 | -0.15 | -0.46 | 0.17 |
| Deep | CIRS respiratory | 0.00 | -0.30 | 0.29 | 0.11 | -0.16 | 0.39 | 0.05 | -0.26 | 0.35 |
| Deep | CIRS renal | **0.90***** | **0.53** | **1.27** | **0.53**** | **0.15** | **0.91** | **0.39*** | **0.01** | **0.77** |
| Deep | CIRS endo.met | 0.08 | -0.21 | 0.37 | 0.11 | -0.16 | 0.37 | 0.00 | -0.28 | 0.27 |
| Deep | CIRS hypertension | **0.41**** | **0.14** | **0.69** | 0.19 | -0.08 | 0.46 | **0.39**** | **0.11** | **0.67** |
| Deep | CIRS composite | **0.19**** | **0.06** | **0.32** | 0.10 | -0.03 | 0.22 | **0.15*** | **0.02** | **0.29** |
| Deep | Sleep disorders | 0.28 | -0.01 | 0.57 | 0.20 | -0.06 | 0.47 | 0.22 | -0.06 | 0.50 |
| Deep | CL >30 | 0.19 | -0.24 | 0.61 | -0.10 | -0.50 | 0.30 | -0.32 | -0.73 | 0.08 |
| Deep | CL >12 | -0.05 | -0.33 | 0.23 | -0.15 | -0.41 | 0.11 | -0.19 | -0.46 | 0.09 |
| Deep | APOE4 | -0.20 | -0.51 | 0.11 | -0.11 | -0.40 | 0.18 | -0.16 | -0.47 | 0.14 |

**Supplementary Table 2. Detailed estimates for each risk factor across principal components in the cognitively unimpaired sample with 95% confidence intervals (CI).** Sex was coded as female = 0 and male = 1; thus, negative estimates indicate higher component scores in female compared to male individuals. Abbreviations: comp = component; post = posterior; BGT = basal ganglia & thalamus; PV = periventricular. **P* < 0.05; ***P* < 0.01; ****P* < 0.001.

|  |  | Unadjusted | | | Age-adjusted | | | Fully adjusted | | |
| --- | --- | --- | --- | --- | --- | --- | --- | --- | --- | --- |
| Comp. | Risk factor | β | CI low | CI high | β | CI low | CI high | β | CI low | CI high |
| Post. | Male sex | **0.41**** | **0.13** | **0.70** | 0.22 | -0.03 | 0.47 | 0.19 | -0.10 | 0.48 |
| Post. | CIRS vascular | 0.23 | -0.14 | 0.60 | 0.18 | -0.13 | 0.50 | 0.11 | -0.22 | 0.44 |
| Post. | CIRS cardiac | 0.10 | -0.26 | 0.47 | -0.17 | -0.48 | 0.15 | -0.22 | -0.56 | 0.11 |
| Post. | CIRS respiratory | -0.05 | -0.38 | 0.28 | 0.04 | -0.24 | 0.32 | -0.02 | -0.33 | 0.29 |
| Post. | CIRS renal | **0.51*** | **0.04** | **0.98** | -0.01 | -0.43 | 0.42 | -0.03 | -0.46 | 0.40 |
| Post. | CIRS endo.met | 0.09 | -0.23 | 0.42 | 0.13 | -0.14 | 0.40 | 0.01 | -0.28 | 0.30 |
| Post. | CIRS hypertension | **0.62***** | **0.31** | **0.93** | **0.29*** | **0.01** | **0.57** | **0.40*** | **0.10** | **0.70** |
| Post. | CIRS composite | **0.19**** | **0.06** | **0.32** | 0.08 | -0.04 | 0.21 | 0.07 | -0.07 | 0.22 |
| Post. | Sleep disorders | 0.17 | -0.15 | 0.49 | 0.01 | -0.26 | 0.28 | 0.02 | -0.27 | 0.31 |
| Post. | CL >30 | 0.14 | -0.44 | 0.71 | -0.07 | -0.56 | 0.41 | -0.17 | -0.66 | 0.33 |
| Post. | CL >12 | -0.04 | -0.36 | 0.28 | -0.08 | -0.35 | 0.19 | -0.14 | -0.44 | 0.15 |
| Post. | APOE4 | -0.18 | -0.52 | 0.15 | -0.07 | -0.35 | 0.22 | -0.07 | -0.38 | 0.24 |
| BGT | Male sex | 0.17 | -0.11 | 0.45 | 0.12 | -0.17 | 0.40 | 0.23 | -0.11 | 0.56 |
| BGT | CIRS vascular | 0.11 | -0.25 | 0.47 | 0.10 | -0.26 | 0.45 | 0.18 | -0.21 | 0.57 |
| BGT | CIRS cardiac | -0.13 | -0.48 | 0.22 | -0.22 | -0.57 | 0.14 | -0.25 | -0.64 | 0.14 |
| BGT | CIRS respiratory | 0.00 | -0.32 | 0.32 | 0.02 | -0.29 | 0.34 | 0.07 | -0.29 | 0.43 |
| BGT | CIRS renal | 0.10 | -0.36 | 0.56 | -0.06 | -0.54 | 0.42 | -0.08 | -0.58 | 0.42 |
| BGT | CIRS endo.met | -0.01 | -0.32 | 0.31 | 0.01 | -0.30 | 0.32 | -0.06 | -0.40 | 0.28 |
| BGT | CIRS hypertension | **0.39*** | **0.09** | **0.70** | **0.32*** | **0.00** | **0.64** | **0.50**** | **0.15** | **0.85** |
| BGT | CIRS composite | **0.19**** | **0.06** | **0.32** | 0.04 | -0.11 | 0.19 | 0.10 | -0.06 | 0.27 |
| BGT | Sleep disorders | -0.10 | -0.41 | 0.21 | -0.15 | -0.45 | 0.16 | -0.21 | -0.55 | 0.12 |
| BGT | CL >30 | -0.38 | -0.93 | 0.17 | -0.44 | -0.99 | 0.10 | -0.46 | -1.03 | 0.11 |
| BGT | CL >12 | -0.08 | -0.39 | 0.23 | -0.09 | -0.40 | 0.21 | -0.09 | -0.44 | 0.25 |
| BGT | APOE4 | -0.14 | -0.46 | 0.19 | -0.10 | -0.42 | 0.22 | -0.06 | -0.42 | 0.30 |
| PV | Male sex | -0.16 | -0.46 | 0.13 | **-0.36**** | **-0.62** | **-0.09** | **-0.56***** | **-0.86** | **-0.25** |
| PV | CIRS vascular | 0.16 | -0.21 | 0.53 | 0.12 | -0.21 | 0.45 | 0.18 | -0.18 | 0.54 |
| PV | CIRS cardiac | 0.23 | -0.14 | 0.59 | -0.01 | -0.34 | 0.33 | 0.01 | -0.35 | 0.38 |
| PV | CIRS respiratory | 0.10 | -0.23 | 0.44 | 0.18 | -0.11 | 0.48 | 0.23 | -0.11 | 0.57 |
| PV | CIRS renal | **0.75**** | **0.28** | **1.21** | 0.32 | -0.13 | 0.76 | 0.29 | -0.18 | 0.75 |
| PV | CIRS endo.met | 0.02 | -0.31 | 0.35 | 0.05 | -0.24 | 0.34 | 0.03 | -0.29 | 0.35 |
| PV | CIRS hypertension | **0.56**** | **0.25** | **0.87** | 0.27 | -0.03 | 0.57 | 0.33 | 0.00 | 0.66 |
| PV | CIRS composite | **0.19**** | **0.06** | **0.32** | 0.13 | 0.00 | 0.27 | **0.18*** | **0.03** | **0.33** |
| PV | Sleep disorders | 0.16 | -0.17 | 0.48 | 0.02 | -0.27 | 0.31 | 0.07 | -0.25 | 0.39 |
| PV | CL >30 | -0.22 | -0.80 | 0.36 | -0.41 | -0.92 | 0.11 | -0.45 | -0.98 | 0.09 |
| PV | CL >12 | -0.16 | -0.48 | 0.16 | -0.20 | -0.48 | 0.09 | -0.21 | -0.53 | 0.12 |
| PV | APOE4 | -0.16 | -0.50 | 0.18 | -0.05 | -0.36 | 0.25 | 0.00 | -0.34 | 0.34 |
| Deep | Male sex | -0.06 | -0.33 | 0.21 | -0.20 | -0.46 | 0.06 | -0.11 | -0.40 | 0.18 |
| Deep | CIRS vascular | 0.08 | -0.27 | 0.43 | 0.05 | -0.27 | 0.38 | 0.12 | -0.21 | 0.45 |
| Deep | CIRS cardiac | -0.10 | -0.44 | 0.24 | -0.28 | -0.60 | 0.04 | -0.22 | -0.56 | 0.11 |
| Deep | CIRS respiratory | 0.06 | -0.25 | 0.37 | 0.12 | -0.17 | 0.41 | 0.11 | -0.20 | 0.42 |
| Deep | CIRS renal | 0.43 | -0.01 | 0.87 | 0.11 | -0.33 | 0.54 | 0.10 | -0.32 | 0.53 |
| Deep | CIRS endo.met | 0.00 | -0.31 | 0.30 | 0.02 | -0.26 | 0.30 | -0.09 | -0.38 | 0.20 |
| Deep | CIRS hypertension | **0.52**** | **0.23** | **0.81** | **0.32*** | **0.03** | **0.61** | **0.51**** | **0.21** | **0.81** |
| Deep | CIRS composite | **0.19**** | **0.06** | **0.32** | 0.06 | -0.08 | 0.19 | 0.12 | -0.03 | 0.26 |
| Deep | Sleep disorders | 0.19 | -0.11 | 0.49 | 0.09 | -0.19 | 0.37 | 0.07 | -0.22 | 0.36 |
| Deep | CL >30 | -0.39 | -0.92 | 0.15 | **-0.53*** | **-1.03** | **-0.03** | **-0.58*** | **-1.06** | **-0.09** |
| Deep | CL >12 | -0.22 | -0.52 | 0.08 | -0.25 | -0.53 | 0.03 | -0.29 | -0.58 | 0.00 |
| Deep | APOE4 | -0.24 | -0.55 | 0.08 | -0.16 | -0.45 | 0.13 | -0.16 | -0.47 | 0.14 |

**Supplementary Table 3. Detailed estimates for each risk factor across principal components in the MCI sample with 95% confidence intervals (CI).** Sex was coded as female = 0 and male = 1; thus, negative estimates indicate higher component scores in female compared to male individuals. Abbreviations: comp = component; post = posterior; BGT = basal ganglia & thalamus; PV = periventricular. **P* < 0.05; ***P* < 0.01; ****P* < 0.001.

|  |  | Unadjusted | | | Age-adjusted | | | Fully adjusted | | |
| --- | --- | --- | --- | --- | --- | --- | --- | --- | --- | --- |
| Comp. | Risk factor | β | CI low | CI high | β | CI low | CI high | β | CI low | CI high |
| Post. | Male sex | -0.05 | -0.62 | 0.52 | 0.06 | -0.44 | 0.56 | -0.06 | -0.76 | 0.63 |
| Post. | CIRS vascular | 0.12 | -0.51 | 0.75 | 0.05 | -0.50 | 0.61 | 0.04 | -0.66 | 0.74 |
| Post. | CIRS cardiac | 0.47 | -0.12 | 1.07 | 0.32 | -0.22 | 0.86 | 0.19 | -0.42 | 0.80 |
| Post. | CIRS respiratory | -0.29 | -0.86 | 0.28 | 0.09 | -0.45 | 0.64 | -0.09 | -0.78 | 0.60 |
| Post. | CIRS renal | 0.97 | 0.41 | 1.54 | **0.60*** | **0.01** | **1.20** | **0.82*** | **0.15** | **1.49** |
| Post. | CIRS endo.met | 0.20 | -0.36 | 0.75 | 0.16 | -0.33 | 0.65 | 0.02 | -0.54 | 0.57 |
| Post. | CIRS hypertension | 0.46 | -0.07 | 0.98 | 0.39 | -0.07 | 0.85 | 0.52 | -0.04 | 1.08 |
| Post. | CIRS composite | **0.19**** | **0.06** | **0.32** | **0.26*** | **0.04** | **0.49** | **0.31*** | **0.03** | **0.58** |
| Post. | Sleep disorders | 0.26 | -0.35 | 0.87 | 0.24 | -0.30 | 0.77 | 0.39 | -0.28 | 1.05 |
| Post. | CL >30 | 0.53 | -0.08 | 1.15 | 0.27 | -0.30 | 0.83 | 0.28 | -0.43 | 0.99 |
| Post. | CL >12 | 0.45 | -0.08 | 0.98 | 0.26 | -0.22 | 0.74 | 0.23 | -0.33 | 0.79 |
| Post. | APOE4 | -0.04 | -0.74 | 0.66 | -0.04 | -0.66 | 0.58 | -0.09 | -0.76 | 0.58 |
| BGT | Male sex | -0.40 | -1.11 | 0.31 | -0.37 | -1.10 | 0.35 | -0.20 | -1.11 | 0.71 |
| BGT | CIRS vascular | 0.38 | -0.41 | 1.18 | 0.36 | -0.44 | 1.17 | 0.68 | -0.21 | 1.58 |
| BGT | CIRS cardiac | 0.17 | -0.61 | 0.95 | 0.13 | -0.66 | 0.92 | 0.08 | -0.73 | 0.88 |
| BGT | CIRS respiratory | 0.25 | -0.48 | 0.98 | 0.41 | -0.38 | 1.19 | -0.16 | -1.06 | 0.74 |
| BGT | CIRS renal | 0.73 | -0.04 | 1.51 | 0.75 | -0.13 | 1.63 | 0.59 | -0.35 | 1.53 |
| BGT | CIRS endo.met | **1.00**** | **0.34** | **1.65** | **0.99**** | **0.33** | **1.64** | **0.70*** | **0.00** | **1.39** |
| BGT | CIRS hypertension | 0.24 | -0.43 | 0.92 | 0.23 | -0.46 | 0.91 | 0.66 | -0.07 | 1.40 |
| BGT | CIRS composite | **0.19**** | **0.06** | **0.32** | **0.48**** | **0.16** | **0.80** | **0.54**** | **0.20** | **0.88** |
| BGT | Sleep disorders | **0.93*** | **0.20** | **1.67** | **0.93*** | **0.19** | **1.66** | **1.18**** | **0.39** | **1.97** |
| BGT | CL >30 | **0.94*** | **0.18** | **1.70** | **0.92*** | **0.13** | **1.72** | **1.22**** | **0.37** | **2.07** |
| BGT | CL >12 | 0.57 | -0.10 | 1.24 | 0.54 | -0.15 | 1.24 | 0.76* | 0.06 | 1.46 |
| BGT | APOE4 | 0.40 | -0.48 | 1.29 | 0.40 | -0.49 | 1.30 | 0.28 | -0.60 | 1.16 |
| PV | Male sex | -0.44 | -1.03 | 0.15 | -0.32 | -0.82 | 0.18 | -0.49 | -1.16 | 0.18 |
| PV | CIRS vascular | 0.50 | -0.15 | 1.16 | 0.43 | -0.12 | 0.97 | 0.60 | -0.07 | 1.27 |
| PV | CIRS cardiac | 0.06 | -0.59 | 0.72 | -0.13 | -0.68 | 0.42 | -0.41 | -1.00 | 0.19 |
| PV | CIRS respiratory | -0.51 | -1.10 | 0.09 | -0.07 | -0.62 | 0.48 | -0.62 | -1.27 | 0.03 |
| PV | CIRS renal | **1.04**** | **0.44** | **1.64** | 0.56 | -0.04 | 1.17 | 0.66 | -0.03 | 1.35 |
| PV | CIRS endo.met | 0.26 | -0.33 | 0.85 | 0.22 | -0.28 | 0.71 | -0.03 | -0.58 | 0.52 |
| PV | CIRS hypertension | 0.32 | -0.24 | 0.89 | 0.24 | -0.23 | 0.71 | 0.34 | -0.22 | 0.90 |
| PV | CIRS composite | **0.19**** | **0.06** | **0.32** | 0.20 | -0.03 | 0.43 | 0.11 | -0.17 | 0.39 |
| PV | Sleep disorders | 0.55 | -0.08 | 1.18 | 0.52* | 0.00 | 1.05 | 0.53 | -0.12 | 1.17 |
| PV | CL >30 | **0.79*** | **0.16** | **1.42** | 0.48 | -0.08 | 1.04 | **0.82*** | **0.16** | **1.48** |
| PV | CL >12 | **0.62*** | **0.07** | **1.17** | 0.39 | -0.09 | 0.88 | **0.63*** | **0.11** | **1.15** |
| PV | APOE4 | 0.61 | -0.11 | 1.33 | 0.61* | 0.01 | 1.21 | **0.72*** | **0.10** | **1.35** |
| Deep | Male sex | **-0.84*** | **-1.53** | **-0.14** | **-0.74*** | **-1.40** | **-0.08** | -0.80 | -1.73 | 0.12 |
| Deep | CIRS vascular | 0.01 | -0.81 | 0.83 | -0.05 | -0.82 | 0.71 | 0.18 | -0.80 | 1.15 |
| Deep | CIRS cardiac | 0.17 | -0.62 | 0.97 | 0.01 | -0.74 | 0.76 | 0.25 | -0.60 | 1.10 |
| Deep | CIRS respiratory | -0.26 | -1.01 | 0.49 | 0.13 | -0.62 | 0.88 | -0.39 | -1.33 | 0.56 |
| Deep | CIRS renal | **1.57***** | **0.88** | **2.26** | 1.35 | 0.59 | 2.11 | **1.57**** | **0.70** | **2.43** |
| Deep | CIRS endo.met | 0.42 | -0.30 | 1.14 | 0.39 | -0.28 | 1.06 | 0.22 | -0.55 | 0.99 |
| Deep | CIRS hypertension | -0.05 | -0.75 | 0.65 | -0.12 | -0.77 | 0.53 | 0.27 | -0.51 | 1.05 |
| Deep | CIRS composite | **0.19**** | **0.06** | **0.32** | 0.23 | -0.09 | 0.55 | 0.35 | -0.02 | 0.73 |
| Deep | Sleep disorders | 0.71 | -0.06 | 1.48 | 0.69 | -0.03 | 1.41 | 0.72 | -0.19 | 1.63 |
| Deep | CL >30 | 0.62 | -0.18 | 1.42 | 0.36 | -0.43 | 1.15 | 0.56 | -0.43 | 1.54 |
| Deep | CL >12 | 0.28 | -0.42 | 0.99 | 0.08 | -0.59 | 0.76 | 0.36 | -0.42 | 1.14 |
| Deep | APOE4 | 0.12 | -0.79 | 1.03 | 0.12 | -0.73 | 0.97 | -0.03 | -0.96 | 0.91 |

**Supplementary Table 4. Regression model results including covariates only are shown for prediction of the four principal component scores for the combined sample (CU + MCI).** *P*-values of significant predictors are shown in bold. The additional adjustment for sex did not considerably change the results.

| **Post. Component** | **Estimate** | **SE** | **t-value** | ***P*-value** |
| --- | --- | --- | --- | --- |
| Intercept | -0.0583 | 0.09217 | -0.633 | 0.52774 |
| CIRS hypertension | 0.42614 | 0.1291 | 3.301 | **0.00115** |
| Age | 0.48156 | 0.06093 | 7.904 | **<0.001** |
| Physical Activity | -0.12056 | 0.05751 | -2.096 | **0.03734** |
| Education | -0.02932 | 0.05829 | -0.503 | 0.61554 |
| Current smoking | -0.22513 | 0.16671 | -1.35 | 0.17846 |
| Former smoking | -0.069 | 0.06345 | -1.087 | 0.27822 |
| Alcohol use | 0.03432 | 0.13866 | 0.248 | 0.80476 |
| **Deep Component** | **Estimate** | **SE** | **t-value** | ***P*-value** |
| Intercept | -0.07556 | 0.10191 | -0.741 | 0.45933 |
| CIRS hypertension | 0.38883 | 0.14275 | 2.724 | **0.00704** |
| Age | 0.29882 | 0.06737 | 4.436 | **<0.001** |
| Physical Activity | -0.05481 | 0.06359 | -0.862 | 0.38978 |
| Education | -0.20697 | 0.06445 | -3.211 | **0.00155** |
| Current smoking | -0.26753 | 0.18434 | -1.451 | 0.14831 |
| Former smoking | -0.01295 | 0.07016 | -0.185 | 0.85373 |
| Alcohol use | -0.07858 | 0.15332 | -0.513 | 0.60888 |
| **PV Component** | **Estimate** | **SE** | **t-value** | ***P*-value** |
| Intercept | -0.070784 | 0.098751 | -0.717 | 0.4744 |
| CIRS hypertension | 0.290796 | 0.138325 | 2.102 | **0.0368** |
| Age | 0.467793 | 0.06528 | 7.17 | **<0.001** |
| Physical Activity | -0.006439 | 0.061616 | -0.105 | 0.9169 |
| Education | 0.030206 | 0.062452 | 0.484 | 0.6292 |
| Current smoking | -0.302777 | 0.178623 | -1.695 | 0.0917 |
| Former smoking | -0.025936 | 0.067986 | -0.381 | 0.7033 |
| Alcohol use | 0.144344 | 0.148568 | 0.972 | 0.3325 |
| **BGT Component** | **Estimate** | **SE** | **t-value** | ***P*-value** |
| Intercept | -0.11763 | 0.1108 | -1.062 | 0.28971 |
| CIRS hypertension | 0.49482 | 0.1552 | 3.188 | **0.00167** |
| Age | 0.02522 | 0.07325 | 0.344 | 0.731 |
| Physical Activity | 0.02069 | 0.06913 | 0.299 | 0.76501 |
| Education | -0.18712 | 0.07007 | -2.67 | **0.00822** |
| Current smoking | -0.2759 | 0.20042 | -1.377 | 0.17022 |
| Former smoking | -0.01472 | 0.07628 | -0.193 | 0.84723 |
| Alcohol use | -0.1103 | 0.1667 | -0.662 | 0.50895 |

| **Post. Component** | **Estimate** | **SE** | **t-value** | ***P*-value** |
| --- | --- | --- | --- | --- |
| Intercept | -0.06492 | 0.10286 | -0.631 | 0.5289 |
| CIRS hypertension | 0.39938 | 0.15259 | 2.617 | **0.00978** |
| Age | 0.49589 | 0.07478 | 6.63 | **<0.001** |
| Physical Activity | -0.13869 | 0.0625 | -2.219 | **0.02801** |
| Education | -0.04618 | 0.06708 | -0.688 | 0.49229 |
| Current smoking | -0.18625 | 0.17736 | -1.05 | 0.29534 |
| Former smoking | -0.07878 | 0.07578 | -1.04 | 0.30024 |
| Alcohol use | 0.02539 | 0.16087 | 0.158 | 0.87481 |
| **Deep Component** | **Estimate** | **SE** | **t-value** | ***P*-value** |
| Intercept | -0.05577 | 0.10264 | -0.543 | 0.587703 |
| CIRS hypertension | 0.50647 | 0.15225 | 3.326 | **0.001108** |
| Age | 0.2728 | 0.07462 | 3.66 | **<0.001** |
| Physical Activity | -0.10574 | 0.06237 | -1.695 | 0.092073 |
| Education | -0.19597 | 0.06694 | -2.928 | **0.00395** |
| Current smoking | -0.35997 | 0.17697 | -2.034 | **0.043717** |
| Former smoking | -0.03736 | 0.07562 | -0.494 | 0.622033 |
| Alcohol use | -0.1444 | 0.16052 | -0.9 | 0.369797 |
| **PV Component** | **Estimate** | **SE** | **t-value** | ***P*-value** |
| Intercept | -0.06332 | 0.11228 | -0.564 | 0.5737 |
| CIRS hypertension | 0.32596 | 0.16656 | 1.957 | 0.0522 |
| Age | 0.4394 | 0.08163 | 5.383 | **<0.001** |
| Physical Activity | -0.03904 | 0.06823 | -0.572 | 0.568 |
| Education | 0.06492 | 0.07322 | 0.887 | 0.3767 |
| Current smoking | -0.37022 | 0.1936 | -1.912 | 0.0578 |
| Former smoking | -0.04282 | 0.08272 | -0.518 | 0.6055 |
| Alcohol use | 0.17983 | 0.1756 | 1.024 | 0.3075 |
| **BGT Component** | **Estimate** | **SE** | **t-value** | ***P*-value** |
| Intercept | -0.022686 | 0.119957 | -0.189 | 0.85026 |
| CIRS hypertension | 0.500598 | 0.177941 | 2.81 | **0.00557** |
| Age | 0.07105 | 0.087211 | 0.815 | 0.417 |
| Physical Activity | 0.004323 | 0.072891 | 0.059 | 0.95279 |
| Education | -0.17206 | 0.078229 | -2.199 | **0.02939** |
| Current smoking | -0.314044 | 0.206825 | -1.518 | 0.13103 |
| Former smoking | -0.040834 | 0.088375 | -0.462 | 0.64472 |
| Alcohol use | -0.179031 | 0.187602 | -0.954 | 0.34147 |

**Supplementary Table 5. Regression model results including covariates only are shown for prediction of the four principal component scores for the CU sample.** *P*-values of significant predictors are shown in bold. The additional adjustment for sex did not considerably change the results.

| **Post. Component** | **Estimate** | **SE** | **t-value** | ***P*-value** |
| --- | --- | --- | --- | --- |
| Intercept | 0.09908 | 0.26447 | 0.375 | 0.71006 |
| CIRS hypertension | 0.51885 | 0.276 | 1.881 | 0.0679 |
| Age | 0.38226 | 0.129 | 2.95 | **0.00545** |
| Physical Activity | 0.11452 | 0.18168 | 0.63 | 0.53234 |
| Education | 0.05961 | 0.13094 | 0.455 | 0.65158 |
| Current smoking | -0.65378 | 0.66516 | -0.983 | 0.33205 |
| Former smoking | -0.0773 | 0.13061 | -0.592 | 0.55757 |
| Alcohol use | 0.0772 | 0.30249 | 0.255 | 0.79997 |
| **Deep Component** | **Estimate** | **SE** | **t-value** | ***P*-value** |
| Intercept | -0.3207 | 0.3685 | -0.87 | 0.3898 |
| CIRS hypertension | 0.2661 | 0.384 | 0.692 | 0.4931 |
| Age | 0.4151 | 0.180 | 2.30 | **0.0272** |
| Physical Activity | 0.3029 | 0.2532 | 1.196 | 0.2391 |
| Education | -0.1678 | 0.1825 | -0.919 | 0.3638 |
| Current smoking | 0.6925 | 0.9269 | 0.747 | 0.4597 |
| Former smoking | 0.1063 | 0.182 | 0.584 | 0.5627 |
| Alcohol use | 0.384 | 0.4215 | 0.911 | 0.3682 |
| **PV Component** | **Estimate** | **SE** | **t-value** | ***P*-value** |
| Intercept | -0.28797 | 0.26339 | -1.093 | 0.281 |
| CIRS hypertension | 0.341 | 0.275 | 1.241 | 0.222 |
| Age | 0.56422 | 0.129 | 4.38 | **<0.001** |
| Physical Activity | 0.14192 | 0.18093 | 0.784 | 0.438 |
| Education | -0.04679 | 0.1304 | -0.359 | 0.722 |
| Current smoking | 0.34118 | 0.66243 | 0.515 | 0.61 |
| Former smoking | 0.05182 | 0.13008 | 0.398 | 0.693 |
| Alcohol use | 0.20042 | 0.30125 | 0.665 | 0.51 |
| **BGT Component** | **Estimate** | **SE** | **t-value** | ***P*-value** |
| Intercept | -0.599 | 0.34802 | -1.722 | 0.0934 |
| CIRS hypertension | 0.665 | 0.363 | 1.831 | 0.0751 |
| Age | 0.00755 | 0.17039 | 0.044 | 0.9649 |
| Physical Activity | 0.12799 | 0.23907 | 0.535 | 0.5956 |
| Education | -0.28935 | 0.17231 | -1.679 | 0.1015 |
| Current smoking | -0.38686 | 0.87529 | -0.442 | 0.6611 |
| Former smoking | 0.13659 | 0.17187 | 0.795 | 0.4318 |
| Alcohol use | 0.17694 | 0.39805 | 0.445 | 0.6593 |

**Supplementary Table 6. Regression model results including covariates only are shown for prediction of the four principal component scores for the MCI sample.** *P*-values of significant predictors are shown in bold. The additional adjustment for sex did not considerably change the results.
